# Blood–Brain Barrier Breakdown and Astrocyte Reactivity Evident in the Absence of Behavioral Changes after Repeated Traumatic Brain Injury

**DOI:** 10.1089/neur.2021.0017

**Published:** 2021-08-27

**Authors:** Celeste Dunn, Nasya Sturdivant, Sara Venier, Syed Ali, Jeffery Wolchok, Kartik Balachandran

**Affiliations:** ^1^ Cell and Molecular Biology Program, University of Arkansas, Fayetteville, Arkansas, USA.; ^2^ Department of Biomedical Engineering, University of Arkansas, Fayetteville, Arkansas, USA.; ^3^ Department of Biological Sciences, University of Arkansas, Fayetteville, Arkansas, USA.; ^4^ Neurochemistry Laboratory, Division of Neurotoxicology, NCTR/FDA, Jefferson, Arkansas, USA.

**Keywords:** blood–brain barrier breakdown, mild TBI, neuroinflammation, repeated TBI, traumatic brain injury (TBI)

## Abstract

Repeated traumatic brain injuries (TBIs) cause debilitating effects. Without understanding the acute effects of repeated TBIs, treatment options to halt further degeneration and damage cannot be developed. This study sought to examine the acute effects of blood–brain barrier (BBB) dysfunction, edema, inflammation and behavioral changes after either a single or double TBI using a C57BL/6 mouse model. We examined the effects of one or two TBIs, of either a mild or moderate severity. Double injuries were spaced 7 days apart, and all analysis was performed 24 h post-injury. To examine edema and inflammation, protein levels of glial fibrillary acidic protein (GFAP), S100 calcium-binding protein B, interleukin-6, and matrix metallopeptidase 9 (MMP9) were analyzed. Aquaporin-4 (AQP4) and zonula occludens-1 (ZO-1) were analyzed to observe BBB dysfunction. Ionized calcium-binding adapter molecule 1 (IBA1) was analyzed to observe microglial activation. Rotarod, beam walking, and grip strength tests were used to measure changes in physical behavior post-injury. A sample size of ≥5 was used for all analysis. Double injuries led to an increase in BBB breakdown, as indicated by altered MMP-9, AQP4, and ZO-1 protein expression. Single injuries showed an increase in microglial activation, astrocyte activation, and BBB breakdown. Behavioral tasks showed no significant differences between injured and control groups. Based on our findings, we suggest that behavioral studies should not be used as the sole clinical indicator on brain tissue recovery. Analysis of markers such as IBA1, GFAP, MMP-9, AQP4, and ZO-1 provide valuable insight on pathophysiological response to injury.

## Introduction

Traumatic brain injury (TBI), defined as an alteration in brain function caused by an external force, is a devastating problem across the world today.^1^ Sixty-nine million persons worldwide are estimated to have sustained a TBI each year.^2^ Short term, or acute, effects of TBIs include inflammation, edema, and blood–brain barrier (BBB) breakdown.^3^ Long-term effects include an increased risk of neurological disorders, such as depression, bipolar disorder, and Alzheimer's and Parkinson's diseases.^4–6^ The state of the cerebral tissue post-injury and secondary effects dictates the severity of the lasting effects of TBIs.^3^ After sustaining one TBI, persons become between 2 and 5 times as likely to sustain another injury.^7,8^ Repeated TBIs can increase the risk of these long-term functional deficits as well as long-term alterations in white matter.^9–11^ Despite the known chronic neurodegenerative effects that occur after repeated TBIs, little is known about the mechanisms that contribute to acute inflammatory and pathological mechanisms that lead to these neurodegenerative diseases.

In terms of injury magnitude, mild TBIs make up 70–90% of all reported TBIs.^12^ Those that suffer from TBIs have no clear “return to play” guidelines. Since 2001, >25 grading approaches have been developed that are based mostly on signs and symptoms.^13^ Behavioral measures are a crude tool and may not parallel the molecular changes in the brain. Early behavioral measures assessed in the clinic can overlook fine-grain tissue-level changes that may manifest later.

Previous studies have shown that repeated TBIs spaced 24–48 h apart result in inflammation and behavioral changes in a rodent model.^12–18^ However, McCrea and colleagues reported that there is an increased risk in experiencing a repeat injury 7–10 days after the primary insult.^19^ Unfortunately, the acute effects of repeat injuries, spaced apart 7–10 days, is woefully understudied. Allowing the brain to at least partially recover between injuries may result in far different behavioral and molecular results than previously studied. For this reason, we aimed to observe the effects of a repeated TBI, spaced 7 days apart.

To address the above gaps in research, we investigated the effects of one or two TBIs on downstream molecular and behavioral events using a C57Bl/6 mouse model. We studied the secondary injuries occurring after receiving no TBI, a single TBI, or two TBIs, either at a mild or moderate severity. Our current study spaced the first and second injury apart by 7 days, in the physiological window of susceptibility for receiving a second injury. We aimed to better understand how these injuries lead to acute inflammation, edema, BBB breakdown, and behavioral changes.

## Methods

### Animals

The University of Arkansas Institutional Animal Care and Use Committee approved all procedures involving the use of animals in this study. C57BL/6 male 6-week-old mice (The Jackson Laboratory, Bar Harbor, ME) were maintained on a 12/12-h light-dark cycle and given standard rodent chow and water *ad libitum*. Cages were changed biweekly, and a daily log of room temperature and humidity was maintained. All animals were checked daily for general health, morbidity, and mortality. Mice were randomly assigned to treatment groups.

### Closed-head traumatic brain injury model

**FIG. 1.**
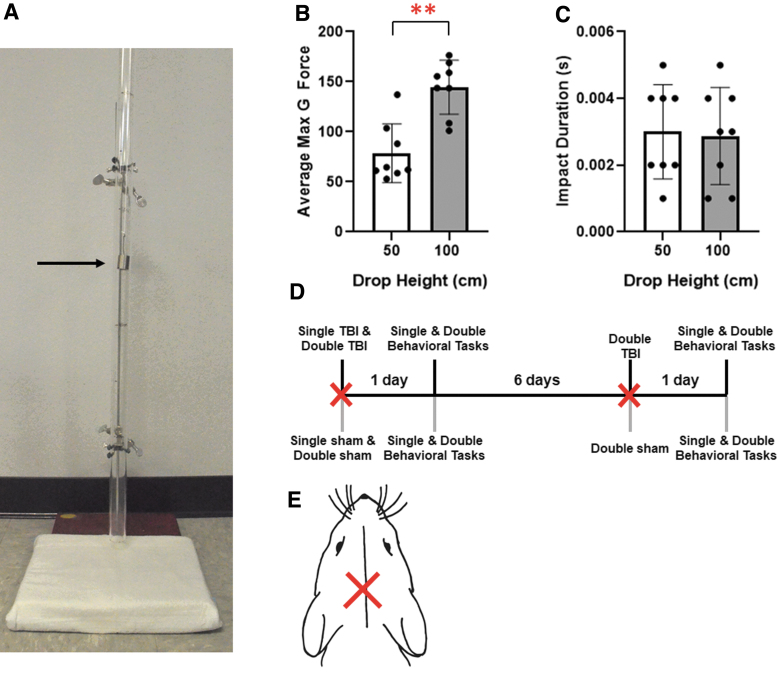
(**A**) An image of the weight-drop apparatus can be seen. A black arrow points to the weight suspended in the acrylic tube. (**B**) Average max G-force upon impact at either 50 or 100 cm. (**C**) Average impact duration from 50 or 100 cm. (**D**) Timeline of events for injury and behavioral testing. **(E)** Location of TBI. Error bars represent SEM. **p < 0.001; *n* = 8. SEM, standard error of the mean.

TBIs were induced using the closed-head weight-drop method.^20,21^ The TBI apparatus consisted of a fixed acrylic tube (2.5 cm inner diameter), elevated over a soft foam pad, and a 50-g weight (2 cm diameter; Fig. 1A). The weight was dropped from different heights, and an attached accelerometer (National Instruments, Austin, TX) measured G-forces on impact, to determine the specific heights for a mild or moderate TBI magnitude. To induce a TBI, mice under isoflurane anesthesia (1–3% in 2 L/min) were placed on the foam pad with their head directly beneath the opening of the acrylic tube. Lidocaine (4%) was administered topically onto the site of injury followed by oral carprofen (5 mg/kg/d) through a dietary supplement (MediGel CPF; Clear H_2_O, Westbrook, ME). Control mice were put under anesthesia and received topical lidocaine application and carprofen, but were not subjected to a TBI. After TBI, mice were monitored regularly for signs of distress. To study the effects of single versus double TBIs, mice either received one TBI or two TBIs, spaced 7 days apart (Fig, 1D). Seven days between injuries was chosen, given that there is evidence of a period of vulnerability 7–10 days after the initial injury.^19^ Twenty-four hours after the final TBI injury, mice were euthanized by 5% isoflurane, followed by decapitation and further analysis, as outlined below.

### Immunohistochemistry

Twenty-four hours after final injury, mice were euthanized, and brain tissue was extracted. Tissue was immediately incubated in 4% paraformaldehyde (PFA) at room temperature for 6 h and then an additional 24 h at 4°C. Brains were then transferred to a solution of 4% PFA/20% sucrose and incubated for 24 h at 4°C, followed by another 24-h incubation in 30% sucrose at 4°C. Brains were embedded in Optimum Cutting Temperature compound (Tissue-Tek; Sakura Finetek, Torrance, CA) and frozen. Brains were coronally sectioned. Standard indirect immunohistochemistry was used to observe the expression of aquaporin-4 (AQP4) and zonula occludens-1 (ZO-1) in brain tissue. Slides were stored at −80°C. Briefly, slides were thawed, hydrated in phosphate-buffered saline (PBS) for 5 min, placed in a sodium citrate buffer (10 mM of sodium citrate, 0.05% Tween-20; pH 6.0), and incubated in a 95°C water bath for 30 min for antigen retrieval. Sections were blocked with 20% goat or donkey serum for 60 min at room temperature. Sections were then incubated with the primary antibody solution overnight at 4°C. Primary antibody solutions were composed of 1:100 ZO-1 (Santa Cruz Technology Inc., Santa Cruz, CA), 1:100 AQP4 (Abcam, Cambridge, MA), 1:1000 cFOS (Novus Biologicals, Littleton, CO), or 1:1000 ionized calcium-binding adaptor molecule 1 (IBA1; Wako, Richmond, VA), diluted in 4% blocking buffer.

Slides were then washed in PBS and incubated in secondary antibodies and 4′,6-diamidino-2-phenylindole for 1 h at room temperature. Slides were washed and protected with ProLong Gold antifade reagent (ThermoFisherScientific, Waltham, MA). Slides were imaged on a confocal or epifluorescence microscope. The number of IBA1-positive pixels was quantified through a custom MATLAB (The MathWorks, Inc., Natick, MA) code, averaging three to six fields per section of tissue. A sample size of 3 was used for each condition, except the control double and moderate single group, which consisted of a sample group of 2.

### Western blot analysis

After euthanasia, the neocortex below the injury site was isolated.^22^ Tissue was then flash frozen and homogenized in radioimmunoprecipitation lysis buffer (Santa Cruz Biotechnology). Homogenates were centrifuged at 13,000*g* for 5 min at 4°C, and the supernatant was collected. Protein concentration was determined using a bicinchoninic acid assay (Life Technologies, Carlsbad, CA). To determine AQP4, ZO-1, and glial fibrillary acidic protein (GFAP) concentrations, Criterion TGX Precast gels (Bio-Rad Laboratories) were run at 200 V for 1 h. Subsequently, the gel was transferred onto polyvinyl difluoride membrane at 100 V for 1 h. After transfer, the membrane was blocked with Odyssey Blocking Buffer (LI-COR, Lincoln, NE) and then probed with one of the following primary antibodies: 1:200 ZO-1 (Santa Cruz Biotechnologies); 1:500 AQP4 (Abcam); 1:500 GFAP (MilliporeSigma, Burlington, MA); or 1:500 glyceraldehyde 3-phosphate dehydrogenase (GAPDH; Abcam) in blocking buffer. GAPDH was used as the loading control. Membranes were then washed and again probed with appropriate secondary antibodies. After washing, protein bands were detected with a LI-COR Odyssey infrared scanner. Band integrated optical density was quantified using ImageJ software (National Institutes of Health, Bethesda, MD).

### S100 calcium-binding protein B, interleukin-6, and matrix metallopeptidase 9 quantification

After tissue lysates were collected, mouse S100 calcium-binding protein B (S100B; LifeSpan Biosciences, Seattle, WA), interleukin-6 (IL-6; ABclonal Technology, Woburn, MA), and matrix metallopeptidase 9 (MMP9; Abcam) were quantified using enzyme-linked immunosorbent assays (ELISAs). All samples were run in duplicate and analyzed, following manufacturer protocols.

### Rotarod test

Before TBI, mice were acclimated to the rotarod for 3 days. For acclimation, they were placed on the rotarod (Ajanta Pharma, Mumbai, India) for 5 min with a linear acceleration of 10 RPM/min for the first minute and a constant velocity of 25 RPM for 4 additional min.^23^ Mice were given a 3-min break between each trial. Mice were tested 24 h after each injury. For testing, mice were placed on the rotarod at an initial speed of 5 RPM with an acceleration rate of 20 RPM/min. Acceleration and timing began 10 sec after mice were placed on the rod. This process was repeated three times. The length of time the mouse was able to remain on the rod was recorded, up to 5 min.

### Grip strength test

Grip strength was tested using a grip strength meter (Harvard Apparatus, Holliston, MA). Mice were tested three times pre- and post-injury.^23^ Mice were held 1 inch from the base of their tails and placed on the grate of the force plate. Once mice had gripped to the force plate, they were gently pulled from the force plate until they released their grip. Mice were given at least 30 sec to rest between each trial, and animals in the double injury groups were reacclimated and retested 24 h before and after their second injury, respectively.

### Beam walking test

Twenty-four hours pre-injury, mice were acclimated to beam walking through three, 30-sec trials on a 1.5-cm square wooden beam (50 cm long).^24^ A black box (15 × 15 × 15 cm) with food was placed at the end of the beam to incentivize mice to cross the beam. Twenty-four hours after each injury, mice were tested through three separate trials. Mice were assessed based on their ability to cross the beams in <30 sec. Additionally, the number of foot slips, inversions, and falls from the beam were recorded per beam per trial. If mice failed to cross the beam in the allotted time, their time to cross was recorded as 30 sec, and the trial was noted as a failure. Double injury conditions were reacclimated and retested 24 h before and after their second injury. After all data were collected, mice were scored based on their ability to successfully cross the beam with no foot slips, inversions, or falls.^15,25^ Each mouse began with 3 points and had the opportunity to score 3, 2, 1, or 0 total points per trial. One point was subtracted for each of the following scenarios: foot slips from the beam; inversions on the beam; and mouse was unwilling to traverse the beam. If the mouse fell completely from the beam, it received a score of 0 for the trial.

### Statistical analysis

All quantitative data were first tested for normality using the Shapiro-Wilk test. Statistical analysis was carried out using two-way analysis of variance for normally distributed data, to study the effect of TBI magnitude and frequency. Non-parametric analysis was used for data that were not normally distributed. All statistical analysis was performed using JMP Pro 15 software (SAS Institute Inc., Cary, NC). Values are expressed as mean ± standard error of the mean (SEM). A *p* value <0.05 was considered statistically significant.

## Results

### Validation of closed-head model

The weight-drop mechanism was validated using methods published previously (Fig. 1A).^21^ The drop height for a mild TBI was 50 cm (78.6 ± 10.3 G-force) whereas the drop height for a moderate TBI was 100 cm (137.4 ± 9.6 G-force; Fig. 1B).^21,27^ Mild and moderate conditions were found to be statistically different (*p* = 0.0003). Duration of impact for both conditions was found to be below the average concussive impact duration, with no statistical significance between mild and moderate impacts (Fig. 1C).^28^ Clinical data are not provided because no differences were observed between treatment and control groups for markers such as righting time. Animals woke up within ∼1 min from being taken off of anesthesia.

### Injury markers

**FIG. 2.**
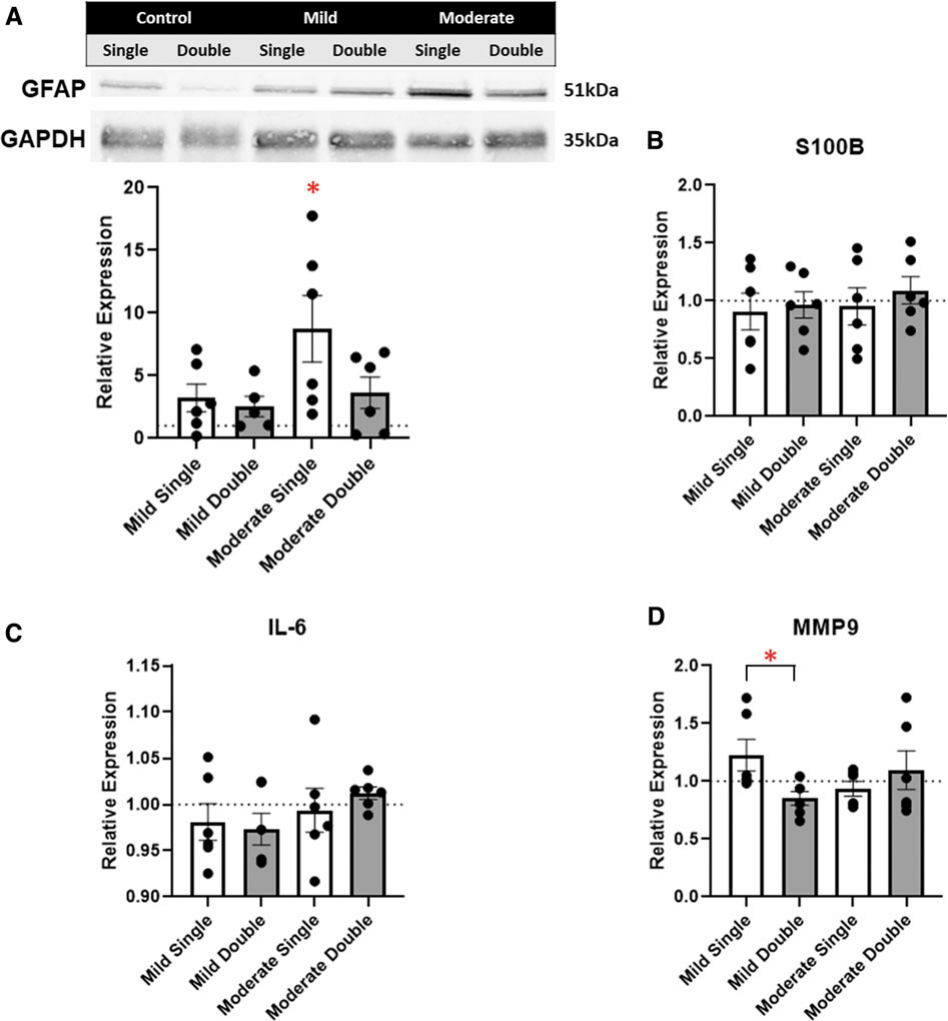
(**A**) Relative expression of GFAP by western blot of the injured tissue was quantified and plotted. (**B–D**) Enzyme-linked immunosorbent assays of the injured cerebral tissue show relative expression of the respective protein. Each sample was compared to its respective control. A dotted line represents the control values. *p < 0.05; *n* = 6. GFAP, glial fibrillary acidic protein.

To examine degree of injury in the different conditions, we analyzed common injury markers in the injured tissue. These markers include the TBI injury marker, GFAP, inflammation markers S100B and IL-6, and the remodeling enzyme, MMP9.^29–33^ All conditions were compared to their respective controls (i.e., either single or double), and relative protein expression was calculated. GFAP, a marker for astrocyte reactivity, was measured through western blot analysis. Overall, all conditions showed an increase in relative GFAP expression fold change (2.54–6.93), with a significant increase in the moderate single condition compared to their respective control (Fig. 2A). IL-6 and S100B expression of injured tissue was observed through ELISAs. IL-6 and S100B expression in isolated tissue varied little compared to respective controls (Fig. 2B,C). MMP9 is an important protease of the extracellular matrix. Lu and colleagues previously reported an increase in MMP9 24 h after TBI injury.^34^ MMP9 expression was measured to gauge remodeling activity of the cortex.^34^ Moderate conditions did not appear to have any significant differences; however, a significant decrease in relative expression of MMP9 in the mild double condition (0.85) was observed compared to the mild single condition (1.23; Fig. 2D).

### Microglial activation

**FIG. 3.**
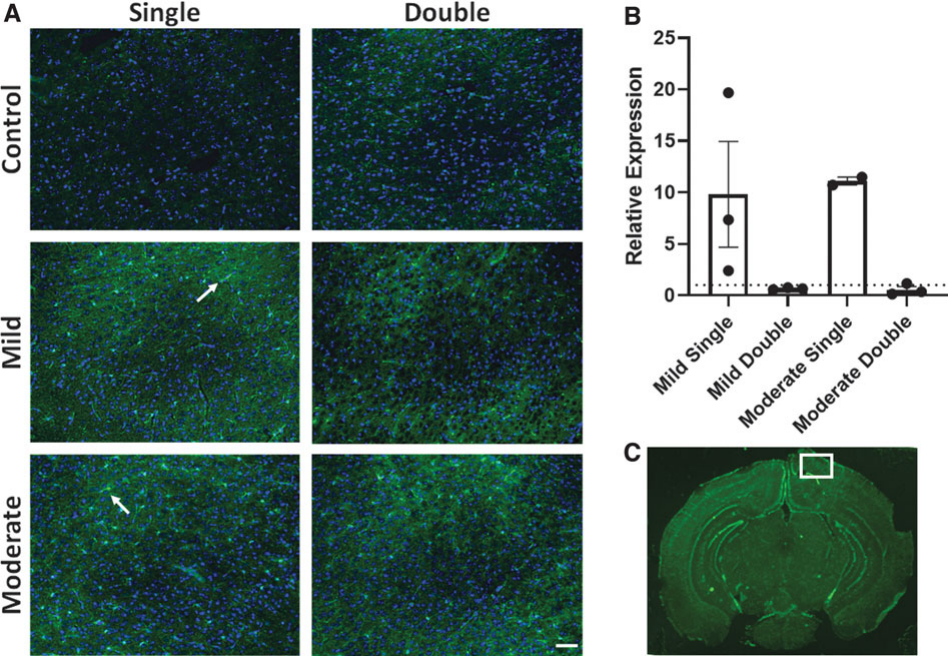
(**A**) Representative IBA1 immunohistochemistry of the injured cortex for the six conditions. (**B**) Relative expression of IBA1 from quantified immunohistochemistry compared to its respective control. (**C**) Area imaged for all immunohistochemistry from the sectioned brains is shown in white. Each sample was compared to its respective control. A dotted line represents the control values. Error bars represent SEM. Scale bar represents 50 μm; *n* = 2–3. IBA1, ionized calcium-binding adapter molecule 1; SEM, standard error of the mean.

IBA1 was used to observe microglial activation.^35^ Immunohistochemistry was performed, and the cortex was imaged (Fig. 3A). Quantification of staining showed an increase in microglial activation in the mild single TBI and moderate single TBI condition compared to the single sham condition (Fig. 3B). Repeated TBI conditions did not appear to change expression levels compared to respective controls.

### Edema/blood–brain barrier markers

**FIG. 4.**
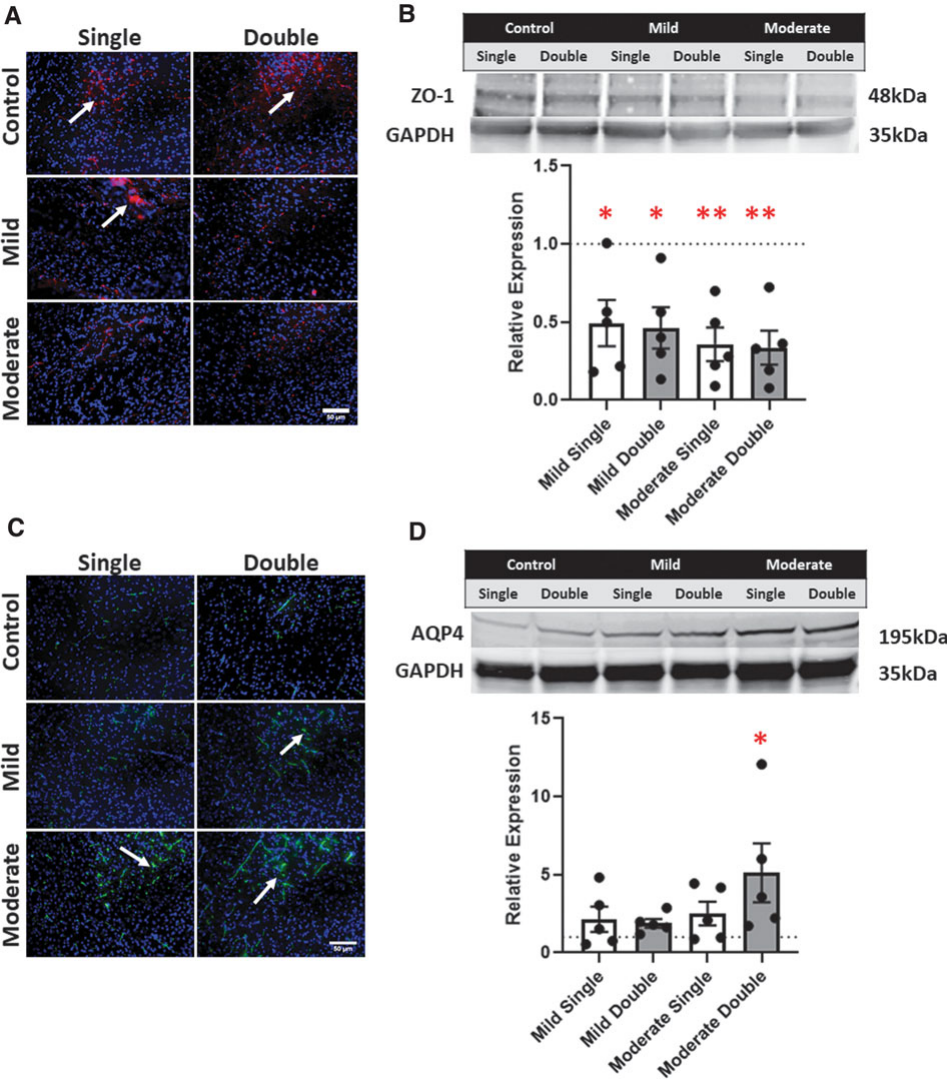
(**A**) Representative ZO-1 immunohistochemistry of the injured cortex for the six conditions. (**B**) Relative expression of ZO-1 by western blot of the injured tissue compared to its respective control. (**C**) Representative AQP4 immunohistochemistry of the injured cortex for the six conditions. (**D**) Representative expression of AQP4 by western blot of the injured tissue. Each sample was compared to its respective control. A dotted line represents the control values. Error bars represent SEM. *p < 0.05; **p < 0.01; *n* = 6. AQP4, aquaporin-4; SEM, standard error of the mean; ZO-1, zonula occludens-1.

ZO-1, a tight junction marker, was measured to determine BBB disruption. A noticeable downregulation in ZO-1 expression after all TBI conditions compared to the respective controls were observed from the ZO-1 immunostains (Fig. 4A). Comparing the mild to the moderate TBI (both in the case of single and double TBI), we observed no difference in ZO-1 expression. Semiquantitative analysis, by western blot, showed a significant decrease in ZO-1 relative expression after all four TBI conditions: mild single condition (0.526); mild double condition (0.491); moderate single TBI (0.380); and a moderate double TBI (0.480; *p* < 0.05 in all conditions; Fig. 4B). AQP4, a water channel protein expressed on astrocyte end feet in the brain, was used to observe BBB dysregulation. AQP4 immunofluorescent stains were performed, and representative images were captured (Fig. 4C). AQP4 expression was upregulated in the mild single condition appeared with respect to single controls. A slight increase in AQP4 expression was also observed in the mild double condition compared to the mild single condition. The largest increase in AQP4 expression, compared to controls, was observed in the moderate single and moderate double conditions. However, we did not observe differences between the single moderate and double moderate TBI conditions.

Western blot analysis was also performed to identify AQP4 expression in the cerebral tissue post-TBI. All conditions experienced an increase in relative expression of AQP4: mild single (2.134); mild double (1.888); moderate single (2.502); and moderate double (5.117; Fig. 4D). A significant increase in relative expression of AQP4 was observed in the moderate double TBI condition (*p* = 0.0371).

### Behavioral analysis

**FIG. 5.**
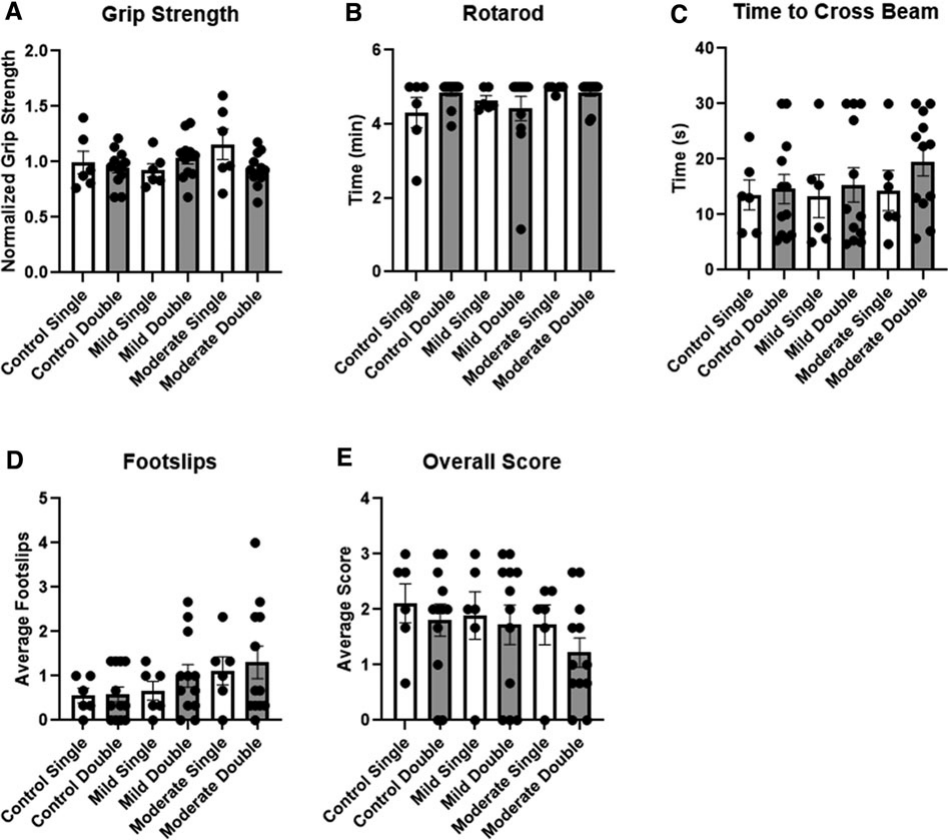
(**A**) Post–grip-strength force was normalized to pre–grip-strength force in the same animal and plotted. (**B**) Time to failure on the rotarod, with a maximum of 5 min is shown. (**C–E**) Time to cross the 1.5-cm beam, number of foot slips, and overall score were plotted. All error bars represent SEM; *n* = 6. SEM, standard error of the mean.

Grip strength was measured before and after injury. Post-TBI grip strength was normalized to the average pre-TBI grip strength (Fig. 5A). No significant findings were noted between conditions. Rotarod testing was used to assess motor function. Time to failure on the rotarod was recorded, up to 5 min. More than half of mice completed the entire length of time on the beam. No significant differences were found between the treatment groups (Fig. 5B). Last, a 50-cm beam was used to measure motor function. Mice were assessed based on time to cross the beam, average foot slips, and overall score. Scores ranged from 0 to 3, where 3 represented mice with no motor issues and 0 were mice with several motor issues. Time to cross the beam was varied within treatment groups, with no significant differences found (Fig. 5C). More foot slips were recorded in the more severe treatment conditions, namely the mild double, moderate single, and moderate double conditions (Fig. 5D). Overall score appeared highly varied and inconsistent across treatment groups (Fig. 5E). No significant differences were observed when conditions were compared to controls for foot slips or overall scores.

## Discussion

Pathophysiology after repeated TBI is largely unknown.^36^ This study aimed to identify differences between single and double injuries through the analysis of injury markers, edema markers, and behavioral responses. To accomplish this, direct impact injuries were administered using an *in vivo* model, spaced 7 days apart, of either a mild or moderate severity. By understanding the pathological and behavioral differences, we sought to better understand the underlying pathophysiology of injured tissue. We initially hypothesized that measurements regarding injury, inflammation, BBB breakdown, and physical tasks would report worse outcomes in the repeated traumatic injuries.

Using the weight-drop method, we have previously shown a significant increase in BBB permeability after a single moderate TBI.^21^ The current results build on our previous work, showing a significant increase in ZO-1 after all single and double TBI conditions and an increase in edema marker AQP4 after a moderate double TBI. GFAP, a common TBI marker, has been shown to be elevated after repeated TBI injuries using a closed-skull impact model.^17^ To study the behavioral effects post-TBI, several studies administer three or more TBIs, with few studies examining two TBIs.^15–17,25,37,38^ For this reason, we aimed to tease out differences in gross motor function between one and two injuries. The purpose of this study was to determine the effects on the BBB, injury markers, and behavioral analysis after one or two TBIs.

GFAP is one of the most widely used clinical markers for TBI.^36^ An increase in GFAP expression indicates astrocyte reactivity and is a main component of glial scar formation.^39^ We demonstrated an increase in cellular GFAP under moderate TBI, consistent with previous literature.^30,36^ Future studies should observe GFAP expression at several time points after repeated TBIs, given that our study was primarily focused on the acute responses after final injury.

IBA1 was used to observe microglial activation after single and double injuries. Interestingly, we found increased activation after single injuries, but not double injuries, regardless of severity. Kane and colleagues reported that repeated TBI did not lead to microglial activation, similar to our findings.^38^ Previous studies have found an increase in IBA1 after single and double injuries; however, these studies observed differences 72 h after impact, not 24 h.^41^ Future studies should observe the microglial response over time after two injuries to better understand how inflammation in the brain responds after being impacted again.

The structural integrity of the BBB was studied through several common markers: AQP4, ZO-1, S100B, IL-6, and MMP-9. AQP4 is a water channel protein expressed on astrocyte end feet. Our results showed a significant increase in AQP4 post-TBI, consistent with current literature.^42^ The increase in AQP4 could suggest cerebral edema after injury, attributable to the dysregulation of AQP4 water channels.^43,44^ ZO-1, a tight junction protein, is commonly observed at lower levels after TBI.^45^ A significant decrease of ZO-1 proteins was observed in all of our conditions, but especially in double TBI conditions. These results further indicate that BBB integrity may be compromised post-injury, and that multiple injuries exacerbate this phenomenon.

S100B, a protein expressed by astrocytes, is a marker of astrocytic activation and BBB leakage.^46^ The level of S100B protein measured in brain tissue correlates with the amount observed in the cerebral spinal fluid (CSF), a commonly measured biomarker for TBI.^47^ Hayakata and colleagues reported, in a previous clinical study, that CSF S100B after a severe TBI was increased within the first 24 h.^48^ Our results did not show any alteration in cortex expression of S100B, though these results were examined in mild and moderate TBIs, as opposed to severe TBI. IL-6 is the first cytokine produced by neurons, glia, and endothelial cells post-injury.^49^ Astrocytic secretion of IL-6 is responsible for increasing BBB permeability within hours after initial insult.^50^ After both single and double injuries, we observed no differences in cortex IL-6 protein values. A previous study reported markedly higher IL-6 levels in post-mortem patients post-injury that led to death.^51^

MMP9, a remodeling protein expressed by endothelial cells, was observed. MMP9 has previously been shown to lead to brain edema post-injury, along with BBB disruption.^52–54^ MMP9 expression peaks at 18 h after a cortical impact injury.^52^ Our results showed relatively consistent MMP9 expression compared to sham animals; however, a significant decrease in MMP9 expression was observed between mild single and mild double conditions. Dynamic MMP9 expression between a single and repeated TBI should be studied further, given that MMP9 has implications in BBB integrity that can lead to edema.

Importantly, our study suggests that behavioral changes should not be the sole factor in determining injury recovery. No significant differences were found across several behavioral parameters, including grip strength, rotarod testing, and beam walking. Several previous studies have shown behavioral changes after repeated TBI; however, repeat injuries were administered 24–48 h apart, whereas our tests subjected mice to injuries 7 days apart.^14–18,25,37^ Although no significant behavioral differences were observed between the control and single and double TBI conditions 24 h post-injury, further studies need to measure behavioral changes days, weeks, and even months after TBI. Monitoring behavioral data, such as sensory deficits, depression, and memory, over longer periods of time after injury is important to better understanding the rate of behavioral changes versus rate of brain molecular changes. These results should be studied to determine a correlation between molecular changes and progression in edema, BBB breakdown, and astrocyte activation. Future studies should also include serum and CSF analysis over time to understand the physiological response to injury and mechanisms of inflammation over the course of recovery between repeated injuries.

## Conclusion

Our validated weight-drop model demonstrated BBB breakdown, as well as an increase in injury markers, after single and repeated TBIs spaced 7 days apart. We observed no behavioral differences. Although there were no measurable behavioral alterations, there were measurable molecular changes to indicate that injury is still present after single and repeated TBIs. Further analysis should be performed regarding multiple TBIs, specifically in regard to behavioral alterations and the rate at which the molecular changes in the brain occur.

## Abbreviations Used

AQP4: aquaporin-4
BBB: blood–brain barrier
CSF: cerebral spinal fluid
ELISAs: enzyme-linked immunosorbent assays
GAPDH: glyceraldehyde 3-phosphate dehydrogenase
GFAP: glial fibrillary acidic protein
IBA1: ionized calcium-binding adaptor molecule 1
IL-6: interleukin-6
MMP9: matrix metallopeptidase 9
PBS: phosphate-buffered saline
PFA: paraformaldehyde
S100B: S100 calcium-binding protein B
SEM: standard error of the mean
TBI: traumatic brain injury
ZO-1: zonula occludens-1
